# Deep UV Microscopy Identifies Prostatic Basal Cells: An Important Biomarker for Prostate Cancer Diagnostics

**DOI:** 10.34133/2022/9847962

**Published:** 2022-09-02

**Authors:** Soheil Soltani, Brian Cheng, Adeboye O. Osunkoya, Francisco E. Robles

**Affiliations:** ^1^Wallace H. Coulter Department of Biomedical Engineering, Georgia Institute of Technology and Emory University, Atlanta, GA 30332, USA; ^2^Departments of Pathology and Urology, Emory University School of Medicine, Atlanta, GA 30322, USA; ^3^Winship Cancer Institute of Emory University, Atlanta, GA 30322, USA

## Abstract

*Objective and Impact Statement*. Identifying benign mimics of prostatic adenocarcinoma remains a significant diagnostic challenge. In this work, we developed an approach based on label-free, high-resolution molecular imaging with multispectral deep ultraviolet (UV) microscopy which identifies important prostate tissue components, including basal cells. This work has significant implications towards improving the pathologic assessment and diagnosis of prostate cancer. *Introduction*. One of the most important indicators of prostate cancer is the absence of basal cells in glands and ducts. However, identifying basal cells using hematoxylin and eosin (H&E) stains, which is the standard of care, can be difficult in a subset of cases. In such situations, pathologists often resort to immunohistochemical (IHC) stains for a definitive diagnosis. However, IHC is expensive and time-consuming and requires more tissue sections which may not be available. In addition, IHC is subject to false-negative or false-positive stains which can potentially lead to an incorrect diagnosis. *Methods*. We leverage the rich molecular information of label-free multispectral deep UV microscopy to uniquely identify basal cells, luminal cells, and inflammatory cells. The method applies an unsupervised geometrical representation of principal component analysis to separate the various components of prostate tissue leading to multiple image representations of the molecular information. *Results*. Our results show that this method accurately and efficiently identifies benign and malignant glands with high fidelity, free of any staining procedures, based on the presence or absence of basal cells. We further use the molecular information to directly generate a high-resolution virtual IHC stain that clearly identifies basal cells, even in cases where IHC stains fail. *Conclusion*. Our simple, low-cost, and label-free deep UV method has the potential to improve and facilitate prostate cancer diagnosis by enabling robust identification of basal cells and other important prostate tissue components.

## 1. Introduction

Prostate cancer (PCa) is the most common extracutaneous cancer malignancy and the second leading cause of cancer-related deaths in men in the United States [1, 2]. Further, biopsy-based studies have shown that more than half of men over the age of 50 harbor some form of prostatic adenocarcinoma or high-grade prostatic intraepithelial neoplasia (HGPIN; a precancerous lesion) [3]. The high prevalence of PCa, combined with (i) early screening (now beginning at 40 years of age using serum prostate-specific antigen (PSA) and/or ultrasound/MRI imaging) and (ii) widespread use of needle biopsy, has made interpretation of small, diagnostically challenging atypical glands a routine part of uropathology practice. Despite active regular screening and application of advanced imaging techniques, cases of false negative and false positive are relatively abundant particularly in challenging cases (especially small foci of glands) and in the nonexpert uropathology setting. (Approximate estimates from different sources suggest a 2-4% false-negative rate and a ~15-35% false-positive rate for prostate cancers [4–12].) For false-positive cases, misdiagnosis of prostate cancer gives rise to unnecessary treatment of healthy patients. For the false-negative cases (which are far more alarming), patients with actual cancer lesions may be left untreated at early stages when interventions can be highly effective. The fact that there is significant diagnostic uncertainty can be psychologically taxing on patients, and the results of overtreatment or undertreatment can have severe consequences for patients’ overall clinical outcome, as well as the economic impact on healthcare systems [5, 13–15].

In routine practice, the diagnosis of PCa is based on histological analysis of prostate tissue sections using hematoxylin and eosin (H&E) stains, and assessment is based on morphological features such as growth pattern, nuclear atypia, and the absence of basal cells [16–19]. However, a number of benign entities closely mimic cancer, making definitive diagnosis challenging, particularly in cases such as small foci of cancer or atypical glands, which as stated above are increasingly common. Therefore, in many cases, it is necessary to use immunohistochemistry (IHC) stains to differentiate cancer from benign entities [20].

Some of the most common IHC stains used for definitive prostate cancer diagnosis include p63 and high-molecular-weight cytokeratin (HMWCK), which selectively label basal cells in prostate tissue [21, 22]. p63 is a homologue of the tumor suppressor gene p53, and HMWCK reacts with the monoclonal antibody keratin 34BE12, both of which are present in the basal layer of prostatic glands [23, 24]. These IHC stains are extremely powerful because the absence of basal cells in glands and ducts is a strong indicator of prostate carcinoma, and the presence of basal cells alone usually precludes a cancer diagnosis for that structure [24]. However, these methods have important limitations. First, IHC antibodies are expensive and require trained personnel and cumbersome procedures for staining. Second, IHC stains do not always react with basal cells, leading to cases where basal cells in benign glands are not stained (negative expression) or are weakly stained [25, 26]. For example, benign lesions such as partial atrophy often result in false-negative stains [24, 27]. Third, there is inherent variability in the uptake of IHC stains that depends on how the tissue is handled, which may also result in weak or no stain expression. Finally, it is not always possible to obtain additional tissue sections for IHC. Thus, there is a critical need for a reliable, robust, and accessible method that can accurately identify basal cells to aid in the diagnosis of suspicious prostate tissue samples.

In this work, we address this important limitation using transmission-based multispectral deep UV microscopy. Recently, deep ultraviolet (UV) imaging has reemerged as an important tool for fast, simple, and reliable label-free molecular imaging based on light attenuation in this spectral region, providing a number of important advantages over conventional optical microscopy modalities (a comparison is provided in the supplemental material). For example, many biomolecules critical to cellular function and the development of diseases have distinct absorption spectral signatures in the deep UV region of the spectrum, which enable high-contrast, quantitative molecular imaging and phenotyping [28, 29]. Further, unlike other label-free molecular imaging methods, deep UV microscopy is fast, offers high spatial resolution owing to its shorter wavelength, and does not require expensive laser systems. (Note that the resolution, r, of a microscope depends on the wavelength, λ, and numerical aperture, NA, of the collecting optics and is given by r=1/2 λ/NA.) Finally, unlike modalities that require fluorescent agents or dyes, deep UV imaging is label-free and quantitative, thus obviating the need for chemical staining [28, 29]. This significantly simplifies laboratory procedures and reduces testing/operational costs and variability. These advantages uniquely position deep UV microscopy as a promising candidate for facile, low-cost quantitative molecular imaging of prostate cancer.

Here, we show that UV spectral and spatial features of basal cells uniquely separate them from other prostate tissue components, enabling stain-free virtual p63 IHC stain, a feat that has yet to be demonstrated despite significant advances in computational imaging and deep neural networks [30–32]. The method proposed here obviates the need for immunohistochemical staining processes and represents a powerful tool that can help pathologists diagnose prostate cancer. Further, given the quantitative nature of UV microscopy, the same method can potentially be optimized for automated computer-aided diagnosis. Finally, the proposed method is nondestructive and preserves the unstained tissue sections. This avoids the need for multiple tissue sections which may not be available and can thus become a particularly critical tool in scenarios where tissue samples are limited, such as in small core needle biopsies.

## 2. Results

### 2.1. Deep UV Microscopy of Prostate Tissue Sections

Details of the multispectral deep UV transmission microscope are provided in Materials and Methods. This system uses attenuation of transmitted UV light through the sample to obtain endogenous molecular information (more details in Materials and Methods). Unlabeled fixed radical prostatectomy tissue samples obtained from formalin-fixed paraffin-embedded (FFPE) blocks were sliced (~5 *μ*m thick) and mounted on quartz microscope slides for imaging. All procedures followed protocols approved by the IRB of our Institution. A filter wheel was used to switch spectral filters at different wavelengths (each with a bandwidth of ~10 nm) to acquire multispectral images from histologically important regions containing structures such as benign tissue, inflammation, stroma, red blood cells, and glands with various grades of prostate cancer. Eighty-seven regions of interest were acquired from 15 patients. Each region was ~1 mm×1.5 mm, acquired with a spatial resolution of ~250 nm. To achieve maximum molecular contrast while minimizing the number of acquisitions, images were taken at four key wavelengths: 220 nm, 255 nm, and 280 nm which correspond to absorption peaks of proteins and nucleic acids [28]. In addition, we included a fourth wavelength at 300 nm which incorporates tissue scattering signatures as an indicator of tissue nanoarchitecture [28, 33, 34].

**Figure 1:**
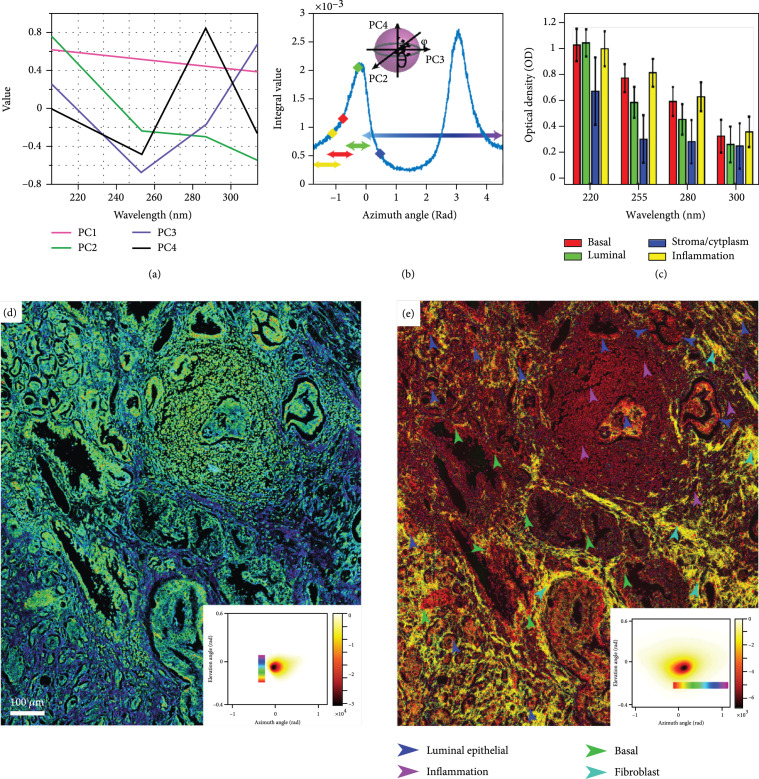
Summary of data processing steps and average spectral data: (a) the 4 principal components resulting from 130 million spectra from representative select regions. (b) Elevation integral of the two-dimensional molecular histogram calculated using projection of multispectral data on principal components 2, 3, and 4. The specified points correspond to the average azimuthal coordinates of inflammation (yellow), basal cells (red), luminal epithelial cells (green), and cytoplasm/stroma (blue). The arrows illustrate the azimuthal angle interval attributed to each component. The inset shows a schematic of coordinate transformation from Cartesian to spherical coordinates. (c) Calculated average spectra from basal cells, luminal epithelial cells, cytoplasm/stroma, and inflammation. (d, e) A representative example of high-contrast molecular colorization using this geometrical representation of the PCs from a prostate tissue region including various components. The insets show the two-dimensional geometrical representation angular distribution histogram of the molecular images using PC 1, 2, and 3 and PC 2, 3, and 4, respectively.

A geometrical representation of principal component analysis (PCA) is applied for further dimension reduction and to enable a visual representation of the spatially resolved spectral signals. PCA is chosen here due to its simplicity and the fact that the PCA inherently maximizes the variance of the data in each principal component, thus yielding a space well-suited to capture both subtle and large molecular changes. In this process, we first selected ~130 million spectra, selected from regions such as gland and stroma components, to calculate the principal components (PCs). Figure 1(a) shows the resulting principal components. Interestingly, the calculated PCs are similar to the absorption and scattering spectra of biological media. Specifically, the spectra of PC1 are similar to the spectral response for tissue scattering with a monotonically decreasing response with increasing wavelength. On the other hand, PC 2 and 4 resemble the absorption spectra of proteins, while PC 3 is similar to the inverted spectra of nucleic acid absorption [28]. However, as we have outlined previously [29], these PCs cannot solely be attributed to these molecular components, and we do not rule out contributions from other molecules.

To leverage the spectral response for molecular imaging, we take the projections of the spectra onto the first three principal components (which contain over 99% of the data variance) and do a coordinate transformation from Cartesian coordinates to spherical coordinates. With this transformation, the shape of the spectrum of each spatial pixel in the image, given by the biochemical composition within that pixel, can be accurately described using only the azimuth (θ) and elevation (ϕ) angles [29]. The radius in spherical coordinates then serves as a relative measure of concentration. Thus, images can be represented in a hue-saturation-value (HSV) color space, with the hue given by the angular coordinates (either elevation or azimuth angle). In practice, multiple representations of the molecular information can be rendered using any combination of three PCs. The proposed colorization method results in two molecular stain maps with contrast for important prostate tissue components such as nuclei, cytoplasm, nerve, stroma, gland concretion, and inflammation as described in [29]. We show two examples of these maps in Figures 1(d) and 1(e).

In Figure 1(d), the elevation angle is used to encode the hue. This optical stain provides clear contrast for nuclei, stroma, and cytoplasm. Since the elevation angle in this representation corresponds to the ratio of the 3rd principal component relative to a combination of the 1st and 2nd PCs, it is expected that this stain provides a strong contrast for nuclei (since the 3rd PC is representative of inverted nucleic acid absorption). Alternatively, in Figure 1(e), we use PC 2, 3, and 4 to allow for more subtle differences in spectral signatures (from weaker PCs and hence biochemical components) to produce appreciable color differences in the optical stains/molecular maps, free from scattering (PC 1) contributions. The resulting images yield nuclear contrast with subtle differences based on cell types (e.g., luminal epithelial, basal, inflammation, and fibroblast nuclei), depicted in different tones of red, while stroma is highlighted in bright yellow. It is worth noting that color variations here primarily reflect differences in the ratio of PC 3 to PC 2 that are attributed to nucleic acid and protein absorption, respectively, which can differentiate between cell nuclei types.

Careful analysis of the optical stains (Figures 1(d) and 1(e)) and the average spectra of different components (calculated from all the patients, Figure 1(b)) suggests that it is possible to utilize spectral differences of biomolecules to uniquely identify various components of prostate tissue, including basal cells that are a strong indicator of benign glands (Figure 1(c)). To this end, we use both geometrical principal component representations applied above (i.e., PC 1, 2, and 3 and PC 2, 3, and 4) in a two-step process to first separate nuclei from stroma and cytoplasm and then categorize different cell types (based on nuclei spectral response). The steps taken to identify all prostate tissue components are detailed in the flowchart in Fig. S2. Briefly, in the first step, we use the elevation direction of the PC 1, 2, and 3 spherical domains to separate nuclei from stroma and cytoplasm. This step is necessary to efficiently separate stroma and cytoplasm structures from cell nuclei (which may have some overlap in the PC 2, 3, and 4 representation). Next, the azimuth direction of the PC 2, 3, and 4 spherical domain is used to differentiate among different nuclei subtypes, as shown in Figure 1(b). Following this two-step process, a slight spectral overlap between stroma, cytoplasm, and nuclei results in an observed “salt and pepper” noise in the final cell nuclear segmented maps. To remove this noise, we apply an area thresholding criteria to eliminate small-area, nonzero clusters of pixels that cannot physically represent cell nuclei.

The analysis above efficiently separates stroma, cytoplasm, and luminal epithelial cells from basal cells and inflammatory cells. However, inflammatory cells and basal cells share some UV spectral overlap and are not always well separated. The spectral overlap can be attributed, in part, to similarities in the chromatin packing structure [35, 36]; however, the shape and anatomical location of these cells differ substantially. Thus, to finally produce label-free optical stains with UV microscopy that faithfully recapitulate p63 and HMWCK IHC, we apply a morphological filtering step. This process effectively identifies the elongated shape of basal cells by computing the gradient of the segmented nuclei map and then applying a dilation procedure followed by intensity thresholding. This procedure (shown schematically in Fig. S2) allows facile separation of the inflammation cells that spectrally overlap with basal cells.

**Figure 2:**
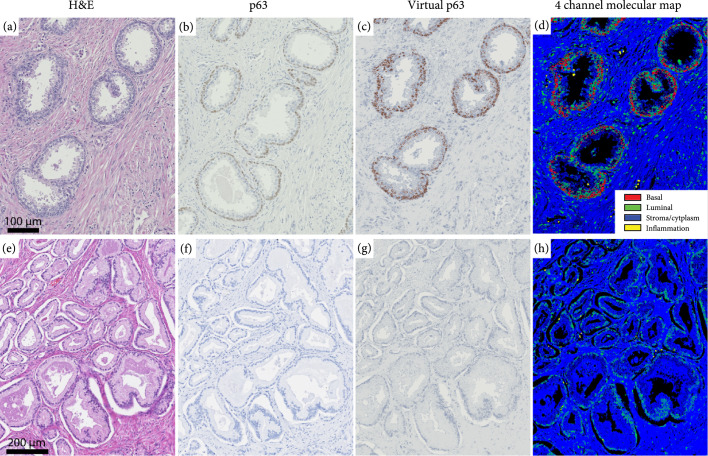
Virtual IHC and 4-channel molecular map colorization for a (a–d) benign and (e–h) prostate cancer tissue. (a, b, e, and f) Show H&E- and p63-stained sections of the same regions (from adjacent sections). As clearly observed, the virtual and stained p63 images are in excellent agreement. Color coding for the 4-channel molecular map in (d, h) is based on the azimuth angle as shown in Figure 1(c) and the cell segmentation procedure described above (red, yellow, green, and blue represent basal cells, inflammation, luminal epithelial cells, and stroma and cytoplasm, respectively).

Finally, we apply two colorization schemes to the nuclear maps. In the first scheme, we seek to recapitulate p63 stains to produce virtual IHC images. Here, basal cells are colorized with a dark brown color while other nuclear subtypes have a dark blue hue and stroma has a light gray shade (as observed in p63). In the second colorization format, we leverage the fact that we can uniquely identify multiple important prostate tissue cellular components to develop a more detailed 4-channel molecular map. Specifically, we encode basal cells in red, luminal epithelial and fibroblast cells in green, inflammatory cells in yellow, and stroma and cytoplasm in blue. These two maps clearly identify the location of the benign gland (given by the presence of basal cells) as well as other important tissue constituents. In Figures 2(a)–2(h), we show two examples of virtual IHCs and 4-channel molecular maps from a benign and cancer region. As clearly highlighted in Figure 2(c), basal cells surround the benign glands with a strong brown hue as seen in a standard p63 stain. Interestingly, in Figures 2(g) and 2(h), there is a lack of basal cells detected with UV microscopy which suggests that the glands correspond to prostatic adenocarcinoma. These findings are indeed in agreement with H&E and p63 stains (from adjacent sections).

Note that with this method, basal cell identification for virtual p63 stains is based on the unique combined absorption spectrum of the nucleic acids, chromatin, keratin content, androgen receptor level, and other intracellular biomolecules [37, 38]. Thus, unlike the actual p63 IHC stain which labels the entire basal cell, this method will only colorize pixels whose absorption profile matches the expected unique spectral signatures of the basal cells. This leads to a “fuzzy” appearance of the basal cells in the virtual p63 stains, compared to the physically stained tissue. Mode conversion methods using deep neural networks [29] may help clean up this appearance and improve the resemblance to real IHC, but this is not necessary as the goal of p63 IHC is to identify basal cells. For this task, even the “fuzzy” virtual stain directly provided by this method is sufficient. Further, given the somewhat limited spectral separation of basal cells to other structures, some small regions may be falsely colorized in brown; however, these regions do not show a clear appreciable structure and do not interfere with basal cell detection nor identification of benign glands.

### 2.2. Application of Virtual IHC in Challenging Diagnostic Cases

The proposed molecular basal cell identification method using label-free deep UV microscopy enables faithful virtual IHC stains, which has the potential to be applied as a powerful tool to aid pathologists in challenging diagnostic cases. Such capabilities can be key when insufficient tissue is available for IHC or when IHC stains show weak or no uptake. Further, adoption of this method can lead to more routine and robust use of basal cell localization in histology analysis since it obviates the need for expensive and time-consuming procedures. In the following section, we apply this method to illustrate its utility in resolving several challenging pathology scenarios in which H&E alone or even in combination with p63 can lead to significant diagnostic uncertainty.

### 2.3. Entrapped Benign Glands

**Figure 3:**
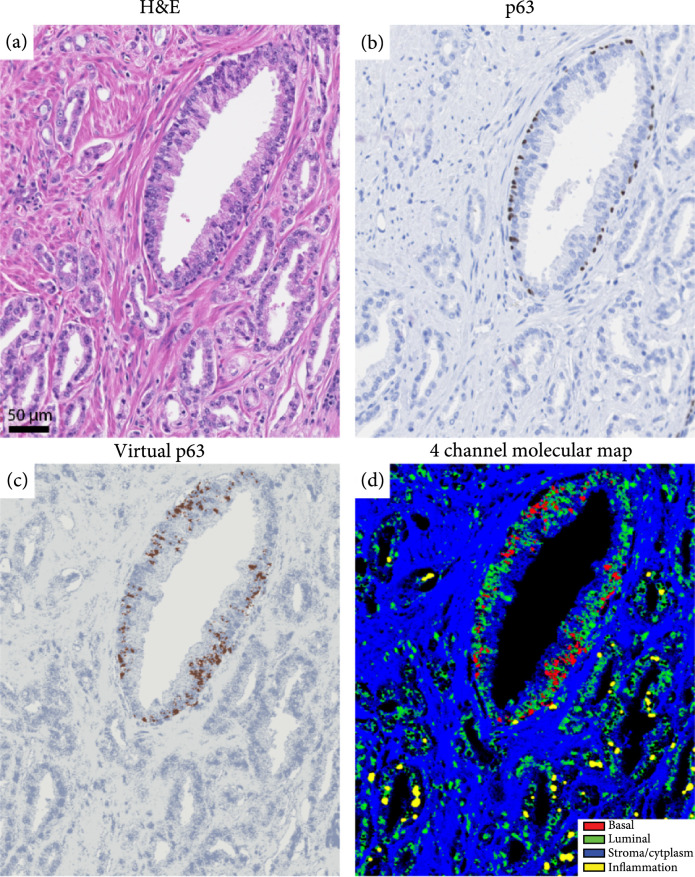
Virtual IHC and 4-channel molecular representation of an entrapped high-grade PIN gland. The existence of basal cells clearly identifies the entrapped high-grade PIN gland with adjacent cancer glands. H&E and p63 stains of the same region are shown for comparison.

In most prostate cancer lesions, there are some entrapped benign glands that do not show any sign of malignancy but are challenging to identify. The importance of entrapped benign gland identification arises from two aspects: First, in some cases, the prevalence of entrapped benign glands can be indicative of adjacent invasive cancer. Second, entrapped benign glands along with colonization by intraductal carcinoma are two possible explanations for residual basal cells [39, 40]. Thus, it is important to identify entrapped basal cells in a cancer region. Our proposed method allows the identification of entrapped benign glands in a cancer region free of any stains. In Figure 3, we show an example of virtual IHC of an entrapped benign gland along with H&E, p63, and the 4-channel molecular image of the same region. Specifically, Figure 3 shows a benign gland with a few basal cells that are surrounded by cancer glands (Gleason Grade 3 and 4). Identification of entrapped benign glands, especially when they lack papillary infoldings and have fewer basal cells, is challenging; thus, IHC imaging is critically important in cases like this. Our proposed UV imaging method is capable of identifying basal cells (and therefore benign glands) only from molecular signatures without the need for extra slices for IHC staining and shows excellent agreement with p63 IHC.

### 2.4. Basal Cell Hyperplasia

**Figure 4:**
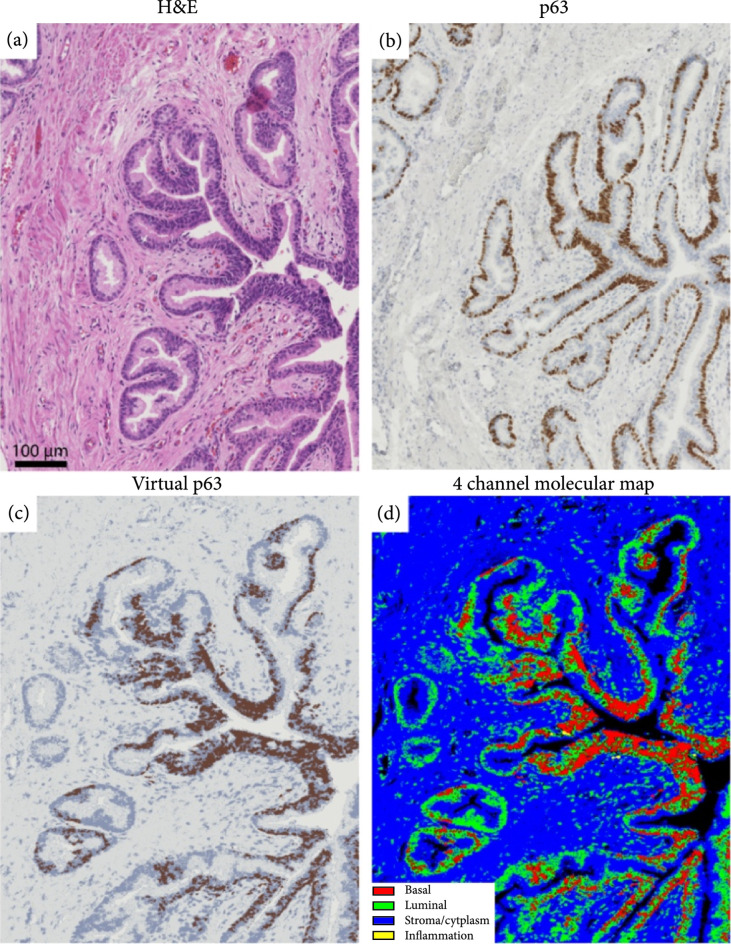
Virtual IHC and 4-channel molecular representation of benign prostate glands with basal cell hyperplasia.

Prostatic epithelium in humans consists of three components: basal cells, luminal epithelial cells, and neuroendocrine cells. Basal cells are slightly smaller than luminal epithelial cells with an exiguous cytoplasm. In normal prostate glands, basal cells represent up to 10% of the cell bodies. But basal cell proliferation in the prostate gland exhibits a wide morphologic continuum ranging from focal basal cell hyperplasia (BCH), in the setting of nodular hyperplasia, to florid adenoid basal cell tumor (ABCT). These diverse proliferations have been referred to by many names including fetalisation of the prostate, embryonal hyperplasia, basal cell tumor, basal cell adenoma, basaloid carcinoma, and adenoid cystic carcinoma. Among them, benign prostatic basal cell hyperplasia (BCH) is a common benign mimic of adenocarcinoma that is challenging to diagnose [41–43]. In many of these cases, BCH requires the use of an IHC panel to differentiate benign BCH from adenocarcinoma. Figure 4 shows an example of benign basal cell hyperplasia, which consists of two types of glands: large gland with few layers of basal cells and few small crowded acini glands. Both of these cases are mimics of adenocarcinoma and are challenging to distinguish from cancer using only H&E-stained tissue slides. This issue is particularly important if we consider the abundance of BCH in prostate tissue. For example, in our existing data set, there are 7 BCH cases (out of 15 patients) with clearly large prostatic glands with two or more layers of basal cells, occasionally protruding in the acinar lumen or small gland basal cell proliferation. This relatively high abundance of cells typically calls for an extra tissue section for IHC staining to obtain a firm diagnosis which adds to the complexity and costs of the diagnosis. Deep UV microscopy can aid in this process by obviating the need for the expense and time-consuming IHC stain, while still providing IHC images that look nearly identical to standard p63 stains (as shown in Figures 4(b) and 4(c)).

### 2.5. Weak or Negative p63 Expression and Atrophy

**Figure 5:**
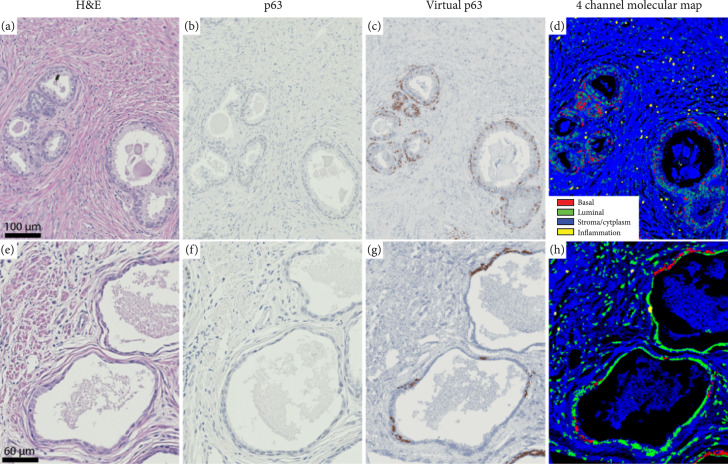
Virtual IHC and 4-channel molecular representation of (a–d) crowded small foci of benign glands with negative p63 expression and (e–h) two cystic atrophic glands with negative p63 expression. Cystic atrophic glands have very few basal cells and might be misinterpreted as cancer. (c–g) Virtual p63 images from deep UV microscopy clearly show the basal cells while the p63 IHC fails to stain them. H&E and p63 stains from adjacent sections are shown for comparison.

One of the most challenging scenarios in prostate cancer diagnostics is when basal cell markers have weak or negative staining. In such cases, it is possible that the lack of staining may be interpreted as malignancy and result in a false positive/overdiagnosis [25, 26]. This is especially important and misleading for small foci of atypical or partially atrophic benign glands. Multispectral deep UV microscopy allows spectroscopic identification of basal cells, and reliability identifies basal cells even when the IHC stain fails. In Figures 5(a)–5(d), we show an example of several small benign glands with negative p63 expression. This an excellent example of small well-formed glands with negative p63 which can be misdiagnosed as Gleason Grade 3 glands with H&E and p63. Indeed, upon close inspection, an expert uropathologist can identify basal cells here with H&E based on the cells’ typical location on the outer layer of prostate glands next to the prostatic stroma and their slightly smaller and/or more elongated shape compared to luminal epithelial cells. Basal cells also exhibit higher hematoxylin stain uptake which gives them a darker blue/purple appearance compared to the relatively pale benign luminal epithelial cells. Nevertheless, less experienced pathologists and/or more difficult cases would require IHC for a more definitive diagnosis—this is why basal cell markers like p63 IHC are so important and used widely for prostate cancer diagnosis. Thus, weak or negative basal cell uptake of p63 is a significant limitation of this stain, which as shown here can be readily addressed by label-free deep UV microscopy.

Figures 5(e)–5(h) show a different example of a “cystic” atrophic gland which also contains basal cells with negative p63 expression. Atrophy is a common benign mimicker of prostate cancer that may occasionally be misdiagnosed. Most of the atrophic glands have only a few distorted basal cells [9, 44, 45]. Here, basal cells can be identified by an expert uropathologist by looking for relatively elongated, dark nuclei that are surrounding luminal epithelial cells and are not within the stroma. But in general, detection of atrophic benign glands becomes especially challenging if the basal cell markers fail to highlight them, as is the case here. Nevertheless, as demonstrated in Figures 5(g) and 5(h), multispectral deep UV microscopy can clearly identify basal cells, independent of the chemical reactivity of basal cells with the stain.

## 3. Discussion

In this study, we have introduced multispectral deep UV microscopy as a fast, cost-effective, and efficient molecular imaging tool that can identify critical structures within prostate tissue sections, including basal cells, without the need for any exogenous labels or dyes. The system also does not require expensive laser systems or complex optical equipment and can potentially be manufactured out of low-cost components (<$5,000 total). The quartz slides used in this work can be replaced by low-cost UV transparent polymers/plastics. Our methodology applies a geometrical representation of principal component analysis to transform the multispectral data into two domains that map out different constituents of prostate tissue. The first domain is dominated by scattering and allows the separation of nuclei from stroma and cytoplasm. The differences in scattering signatures arise from tissue nanoarchitecture alterations [34, 46–48]. The second domain, which is calculated by removing scattering-dominated PC1, uses absorption variations from tissue components (prominently proteins and nucleic acids) and allows differentiation of main gland constituents, including basal cells, inflammation, luminal epithelial cells, stroma, and cytoplasm.

Among all the separated tissue components, the unique identification of basal cells is of critical importance for prostate cancer diagnosis because the lack of basal cells in a prostate gland is a strong indication of prostate cancer development. In a number of cases, identification of basal cells using only H&E stains may be quite challenging. This issue necessitates the utilization of IHC stains to label basal cells, but IHC is expensive, requires additional tissue slice, and does not always successfully stain basal cells. Here, for the first time to our knowledge, we have developed a novel approach that uniquely identifies basal cells without the need for stains or labels using multispectral deep UV microscopy. We hypothesize that the spectral difference between basal cells and other subtypes of cells arises from parameters such as chromatin packing, keratin content, and androgen receptor level, among other factors [37, 38]. Identifying the specific molecular factors that enable unique basal cell identification will be part of our future work.

We have also shown that UV microscopy can be applied to directly produce high-fidelity virtual p63 IHC images. This has a number of important implications. For example, there are cases where extra tissue slices are not available for IHC staining or IHC stains have weak or no expression (or there might be patchy staining) due to staining procedure flaws or intrinsic basal cell uptake failure. Also, the growing demand for routine, fast, and robust basal cell localization in histology requires the development of methods that are free of any expensive and time-consuming procedures. In all these cases, deep UV microscopy allows fast, cost-effective, and accurate access to basal cell content and virtual p63 maps.

One of the most important implications of using multispectral deep UV microscopy for basal cell identification is detection of benign mimics of prostate cancer without utilizing any staining procedures. There are a class of benign mimickers of prostatic adenocarcinoma that specifically require the detection of basal cells to confirm a benign diagnosis. These cancer mimics include (1) atrophy and its variants, including simple atrophy, partial atrophy, and postatrophic hyperplasia, (2) crowded small gland proliferation with small or no atypia, (3) adenosis, (4) hyperplastic and metaplastic lesions, (5) seminal vesicle epithelium, (6) paraganglia, (7) urothelial metaplasia, (8) squamous metaplasia, and (9) sclerosing adenosis [9]. In all these cases, using multispectral deep UV microscopy can facilitate the identification of basal cells, significantly reducing diagnosis costs and uncertainty.

**Figure 6:**
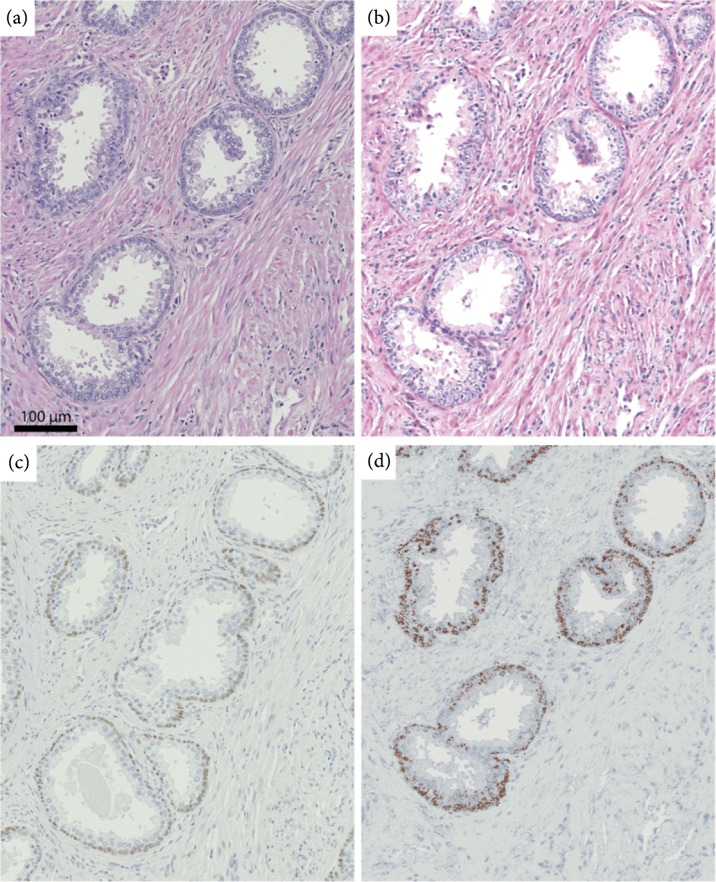
Virtual H&E and p63 stains of a representative prostate region with foci of small benign glands (mimics Gleason Grade 3 prostatic adenocarcinoma). (a) H&E and (b) virtual H&E rendered using a deep learning model as described in Ref. [29]. (c) p63 and (d) virtual p63 rendered using a geometrical representation of PCA.

It is important to highlight that in our previous work [29], we have shown that label-free UV imaging can also produce high-fidelity H&E images. With the added ability to produce virtual IHC images from the same multispectral UV data, as shown in this work, UV microscopy effectively obviates the need for much of the gold-standard tissue processing being done today. In fact, deep UV microscopy can be used to generate a multistain panel (H&E, p63 IHC, and various “optical” stains) at no extra cost while also preserving the unstained tissue sections (note that with the short exposure times of <100 ms per image and low power illumination used <40 *μ*W, no damage is observed in the fixed tissues). To illustrate this important feature, Figures 6(b) and 6(d) show an example of an unstained prostate tissue section from a region with foci of small benign glands imaged with deep UV microscopy and virtually stained to appear like H&E and p63. Again, both of these images are generated from the same multispectral deep UV microscopy data and show excellent agreement with adjacent tissue sections stained with H&E (Figure 6(a)) and p63 IHC (Figure 6(c)). The same unstained tissue section shown in Figures 6(b) and 6(d) was then stained with H&E (Fig. S4) and shows excellent agreement with the deep UV virtual H&E stain. This also demonstrates that this method does not damage or alter the unstained tissue sections and that the tissues can indeed be used for additional tests after UV imaging.

Other label-free methods have been proposed for virtual H&E staining based on deep learning (e.g., using phase contrast [49], autofluorescence [50], and brightfield [51]), but to our knowledge, deep UV microscopy is the only approach that has demonstrated the ability to provide both H&E and highly specific p63 IHC simultaneously (or just label-free p63 IHC virtual stains). Further, unlike virtual H&E staining, the proposed virtual IHC stain does not rely on deep learning methods which, while promising, have their own unique challenges [29, 50–52]. For instance, deep learning requires a lot of matched labeled data in order to train a conversion model, thus inherent artifacts in the images—like weak or negative p63 expression and faded staining—can be learned. Thus, as shown here, our approach which directly uses endogenous scattering and absorption signatures has the unique potential to not just duplicate IHC but actually improve it.

In our previous work [29], we have shown that the endogenous UV molecular information can serve as a personalized continuous prostate cancer biomarker to help grade disease. However, this method measures relative molecular shifts of cancerous glands relative to benign glands from individual patients and thus relies on an expert pathologist to help identify benign prostate glands as Reference [29]. In this current work, we show that multispectral deep UV imaging can also aid in this process, as benign prostate glands can be easily identified based on basal cell content. This is an important finding because it can potentially result in automatic cancer detection and grading. Further, prostate tumor invasion is believed to be a multistage process, progressing sequentially from benign to malignant with associated invasion. Part of the process leading to invasion is triggered by the overproduction of proteolytic enzymes primarily by cancer cells, which leads to focal glandular basal cell disruption [53–55]. Since UV microscopy uses molecular information to determine the presence or absence of basal cells, it could conceivably facilitate the detection of prostate cancer at a relatively early stage.

It is important to emphasize that our proposed method is simply a brightfield transmission microscope with deep UV illumination and can thus be integrated into the current clinical workflow much like modern whole slide digital scanners. Indeed, we use quartz slides in this work, but tissues can be mounted on low-cost UV-transparent polymers/plastics. Further, estimated total acquisition times with our system for a 10 mm×10 mm slide is currently about 1 min per wavelength which yields a throughput of 15 slides/hr with 4 wavelength acquisitions (commercial visible digital slide scanners have a throughput of 20-80 slides/hr). The computational time for image conversion is also negligible (<1 min for a whole slide). With multiplexing and further improvements in automation, higher scanning rates may be achievable. Note, however, that with a single acquisition of our deep UV system, multiple virtual stains can be obtained, including H&E, p63 IHC, the 4-channel molecular map, and other unique optical stains as shown in Figure 1. Moreover, given the rich molecular content and high-resolution structural detail of the deep UV images—along with the increasing power of computational deep learning methods—it is also possible that this method may provide a path towards mimicking other important molecular stains or providing additional unique insight. Thus, deep UV microscopy may ultimately be much faster and lower cost than current procedures required to obtain the same information.

In conclusion, we have demonstrated that multispectral deep UV microscopy is a novel quantitative tool that allows automatic detection of basal cells with high fidelity and free of any stain or chemical processes. This method, along with our previous label-free virtual H&E staining with UV microscopy, has profound implications in aiding pathologists in the diagnosis of prostate cancer. This entire label-free pipeline may also be used for automatic cancer detection and grading in the future. The method is fast, low-cost, and simple and provides subcellular resolution and can be used as a possible tool along with standard methods (such as H&E) to increase diagnostic confidence, accuracy, and reproducibility.

## 4. Materials and Methods

### 4.1. Deep UV Multispectral Microscopy Setup

The deep UV transmission images were acquired using a microscopy system that consists of a plasma-driven broadband light source (Energetiq, EQ-99X). The light source provides a continuous spectrum from 200 nm to 2 *μ*m. The output light from the source is relayed to the sample using an off-axis parabolic mirror (Newport). A long-pass dichroic mirror is used to filter out the wavelengths of light above ~550 nm to remove unnecessary exposure. For each region of interest, a multispectral data cube is captured using bandpass filters centered at 220, 255, 280, and 300 nm (all with a bandwidth of ~10 nm). A filter wheel is used to change the imaging wavelength of the system. A 0.5 N.A. UV objective (Thorlabs LMU-40X-UVB) is used to collect the transmitted light, and a biconvex (f=150 mm) lens is used to relay light onto a UV camera (pco.ultraviolet). A schematic of the setup is shown in Fig. S1. For each acquisition, the camera integration time was set to ~100 ms to capture a field of view of about ~170 μm×230 μm. The resolution of our system is ~250 nm. In this work, we studied regions that were comprised of 81 tiles in the form of a 9 by 9 mosaic image. To enable reliable stitching, each tile has ~15% overlap with its neighbors. The final resulting region is approximately ~1 mm×1.5 mm.

### 4.2. Sample Collection and Preparation

We collected formalin-fixed paraffin-embedded blocks (FFPE) from radical prostatectomy specimens from 15 prostate cancer patients. The patients had not received any neoadjuvant therapy prior to radical prostatectomy. Next, thin slices (~5 microns thick) of the tissue blocks were mounted on quartz slides and were deparaffinized by incubating the slides in xylene bath for 5 minutes. The samples were then placed in 95% ethanol for 3 minutes to remove xylene and washed with deionized water. For each region of interest, we used three adjacent slices. One section was used for UV imaging, and a second section was stained with H&E and imaged with a bright field microscope. In addition, a third section was stained with p63 stain to identify basal cells in case of benign gland presence.

All tissues were deidentified from archived tissue blocks at our Institution (n=15) or a commercial vendor (Biomax) (n=5). This work is conducted under an IRB exempt protocol (H16343).

### 4.3. Data Processing

To study the molecular content of the imaged tissue slides, different wavelengths in each captured multispectral data cube were registered in MATLAB (MathWorks) environment. Next, in order to have a single wide-field UV image, we used an image stitching code (MIST) [56] developed by the National Institute of Standards to stitch the 81 tiles, captured separately.

To calculate the principal components (PCs) of the multispectral prostate tissue images, we selected 90 regions that yielded approximately ~130 million spectra which represented all biologically important structures in prostate tissue. Next, we performed PCA in MATLAB to calculate the 4 principal components of the selected regions.

To separate different components of tissue using molecular signatures, first, we calculated the projections of the multispectral UV data on PC 1, 2, 3, and 4, respectively. Next, we converted the resulting projection vectors (Proj 1, Proj 2, and Proj 3) and (Proj 2, Proj 3, and Proj 4) from Cartesian coordinates to spherical coordinates (azimuth (θ), elevation (ϕ), and radius I), where Prj i represents the projection of UV data on PCi. In the first step of separation, we use the elevation component of PC 1, 2, and 3 representation to separate the nuclei from stroma and cytoplasm. Next, we used the azimuthal component of the PC 2, 3, and 4 representation to separate different species of cells. At this step, the final separated maps, especially the separated basal cell map, contain residual salt and pepper noise as well as misidentified cells from spectrally overlapping molecular species such as inflammation and luminal epithelial cells. To remove the noise, we used a dilation followed by an area filtering step that voids small nonzero pixels. At this step, there are some inflammation cells that are still present in the basal cell map. Here, we used morphological features of basal cells to remove the residual misidentified inflammation. The Prewitt gradient [57] of the separated nuclei exhibits a larger magnitude for most of the basal cells compared to other molecular species. We utilize this feature to separate basal cells from misidentified inflammation. To this end, we have calculated the Prewitt gradient of the nuclear image and apply an intensity threshold followed by a Gaussian filter. This step produces a dilated mask of approximate locations of basal cells and allows the removal of misidentified inflammation. Finally, to produce a virtual p63 image, we have applied three average representative colors to basal cells, other nuclei, and stroma that allows colorization of the image similar to a p63. In addition, we have generated a false color image for each region by using red (for basal cells), green (for luminal epithelial, fibroblast, and smooth muscle nuclei), blue (for stroma and cytoplasm), and yellow for inflammation.

## Data Availability

The data generated in this study are available upon request from the corresponding author.

## Supplementary Materials

Supplementary Materials Fig. S1: a schematic of the multispectral deep UV microscope. Fig. S2: flowchart of processing steps taken to remove salt and pepper noise and misidentified inflammation cells from basal cell content. Fig. S3: background intensity images at 220, 255, 280, and 300 nm captured for one field of view (~170 μm×230 μm) and rescaled from 0 to 1. Fig. S4: scans of H&E-stained regions after multispectral deep UV imaging. The rescanned regions are corresponding (a) Figure 2(c), (b) Figure 5(c), (c) Figure 5(g), and (d) Figure 3(c) in the main text. It is clear that the tissue slices are not damaged and can be stained for further analysis.
