# Supplementary material for: Deep UV Microscopy Identifies Prostatic Basal Cells: An Important Biomarker for Prostate Cancer Diagnostics
Source: BME Front. 2022 Sep 2;2022:9847962. doi: 10.34133/2022/9847962 (PMC10521648; doi:10.34133/2022/9847962)
Supplement: Supplementary Materials — Fig. S1: a schematic of the multispectral deep UV microscope. Fig. S2: flowchart of processing steps taken to remove salt and pepper noise and misidentified inflammation cells from basal cell content. Fig. S3: background intensity images at 220, 255, 280, and 300 nm captured for one field of view (~170 μm×230 μm) and rescaled from 0 to 1. Fig. S4: scans of H&E-stained regions after multispectral deep UV imaging. The rescanned regions are corresponding (a) Figure 2(c), (b) Figure 5(c), (c) Figure 5(g), and (d) Figure 3(c) in the main text. It is clear that the tissue slices are not damaged and can be stained for further analysis. [file 9847962.f1.docx]

Supplementary Materials for

**Deep UV Microscopy Identifies Prostatic Basal Cells: An Important Biomarker for Prostate Cancer**

Soheil Soltani, Brian Cheng, Adeboye O Osunkoya, Francisco E Robles

*Corresponding author Email: robles@gatech.edu


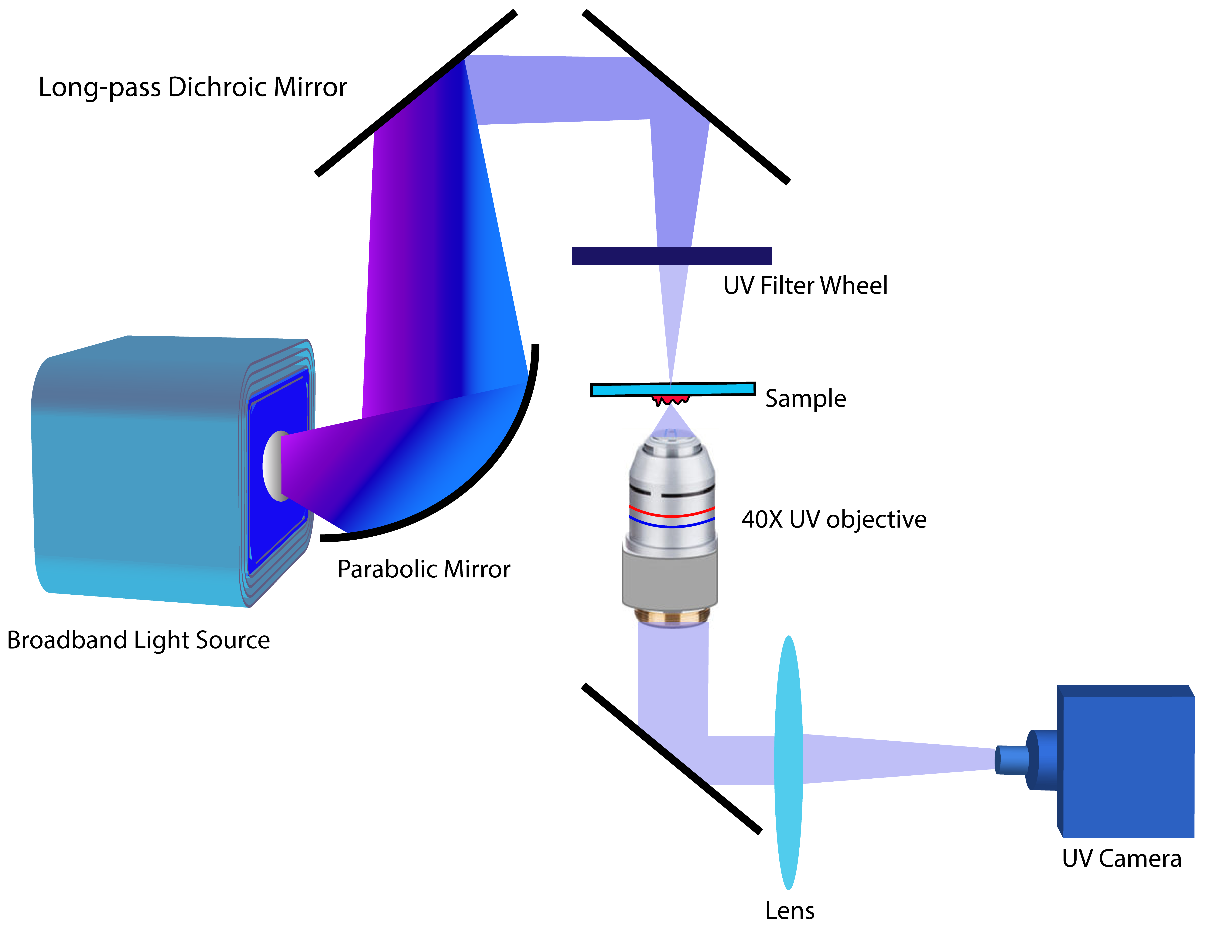


Fig. S1. A schematic of the multi-spectral deep UV microscope. The source provides a broadband output beam (~200 nm to 2000 nm) that is focused on the sample using a parabolic mirror. A dichroic mirror is used to only select deep UV region of the spectrum (200-550 nm). The transmitted light is collected using a 40X UV objective and is relayed on the camera using a biconvex lens


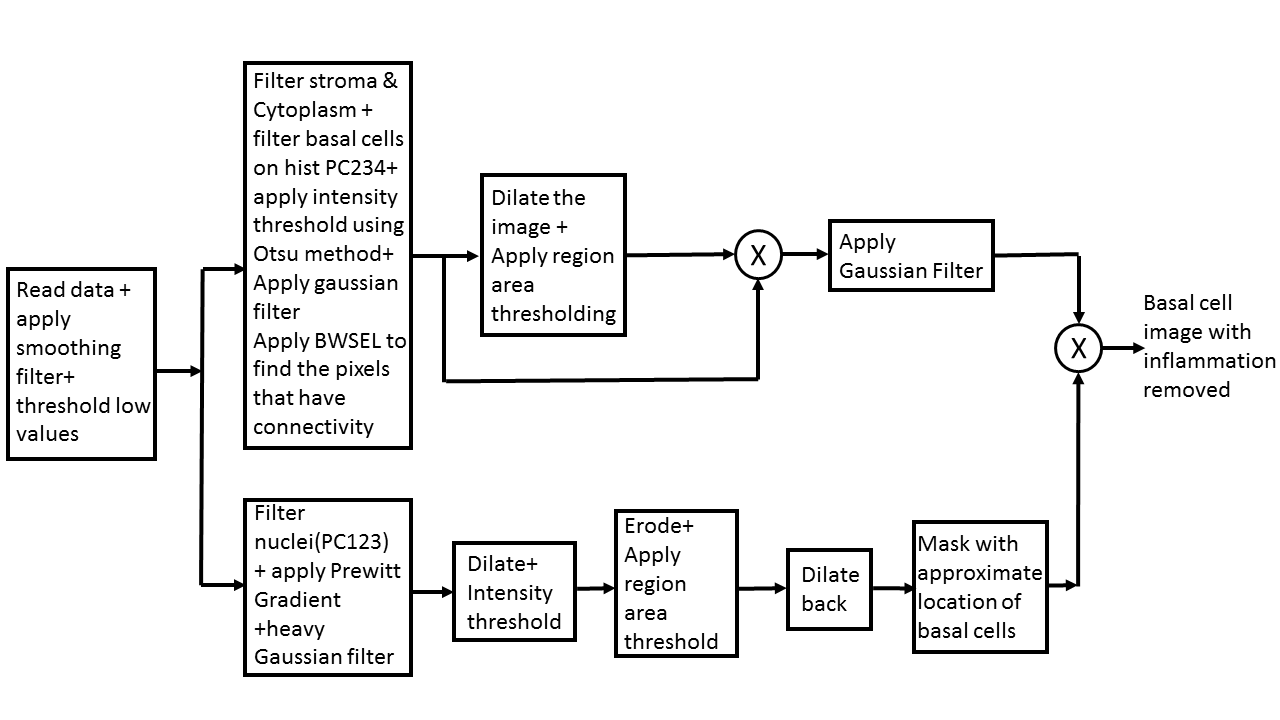


Fig. S2. Flowchart of processing steps taken to remove salt and pepper noise and misidentified inflammation cells from basal cell content.

**Background distribution at different wavelength bands**

The system is designed to provide even illumination across the field of view for all the wavelengths used in the study. Figure S3 shows background intensity images (rescaled from 0 to 1) at different wavelengths, without a sample and light only passing through quartz slide.

As can be seen, the illumination is very uniform. The very small variations in the illumination are eliminated from sample images by normalizing (dividing) by a background region, such as the one shown below.


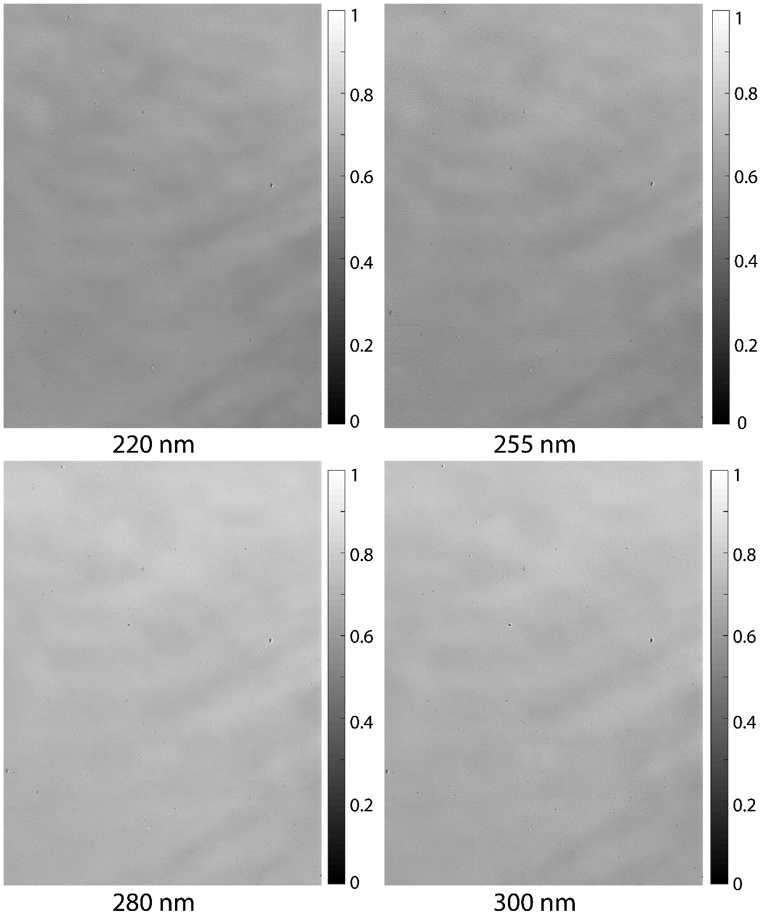


Fig. S3. Background intensity images at 220,255,280 and 300 nm captured for one field of view (~170 μm × 230 μm) and rescaled from 0 to 1.

**Comparison of deep-UV microscopy with other optical imaging methods for molecular imaging tissue sections**

1-Use conventional “visible light” devices: Biomolecules other than hemoglobin and melanin do not absorb in the visible region of spectrum and therefore this range does not provide necessary contrast for molecular differentiation(*1-3*).

2-IR region: Bio-molecules at IR region of spectrum show strong vibrational absorption peaks and can identify many biomolecules. However, devices working at the IR regions of spectrum are expensive, complex and slow. Further since IR wavelengths are relatively longer they do not provide necessary cellular level resolution(*4-7*).

3-UV excited florescence methods: These methods are capable of providing H&E level high resolution histology images as well as IHC imaging however, they require exogenous agents and therefore they are not label free and they are as cumbersome as actual IHC staining methods(*8-10*).

4-Auto-florescent based methods: Auto florescence is used to generate label-free H&E-like images however: (a) auto-florescence intensity differs from patient to patient, (b) the signal to noise ratio is low, and (c) level of endogenous molecular contrast is low(*11-13*).

5- Raman microscopy/spectroscopy: These methods provide rich molecular information and allow differentiation of molecular content. However, Raman scattering is a weak process that requires long acquisition times and signal can easily be obscured by fluorescence. Nonlinear coherent Raman imaging is much faster but systems are complex and expensive(*14-16*).

Finally, most of these optical technologies suffer from complexities in how to integrate into current pathology practice workflows.

On the other hand, label-free multi-spectral deep UV microscopy approach proposed here, using 4 UV wavelengths, shows unique capabilities which overcomes many of the limitations of other methods described above. As shown in this work UV microcopy has the necessary contrast to identify basal cells and uniquely produce virtual IHC stains, as well as virtual H&E and other optical stains. Adding more UV wavelengths may provide additional information that could yield better distinction of different biomolecules but this comes at the cost of more imaging times. The 4 selected wavelengths used here provide sufficient information to translate our images to H&E and p63 IHC, with consistent and unique spectral signatures for basal cells, luminal cells, inflammation cells, and stroma/cytoplasm. Finally, this approach is high-resolution (~300nm), provides rich molecular information, and it is simple and low-cost (~$20k but can be reduced to <$5k; and the UV transparent quartz slides used here could also be replaced by cheap UV transparent polymers). The approach is also widefield with exposures of <100ms per field of view (~170 μm × 230 μm) making it relatively fast.

**Staining tissue slides after UV imaging procedure**

To show that the proposed label free imaging method does not induce any damage and that the UV-imaged sections can be reused for additional procedures, here we show four different regions that we have stained with H&E after performing multispectral deep UV imaging. As clearly observed in Fig.S4 the UV imaging procedure does not damage the tissue and the quality of the stained images are the same any other normal H&E-stained tissue.


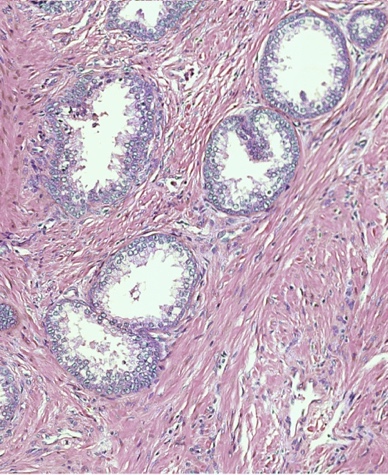

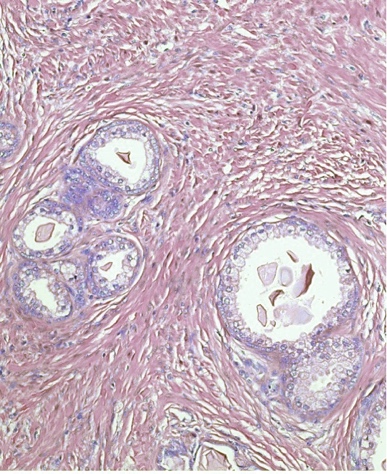


(a) (b)


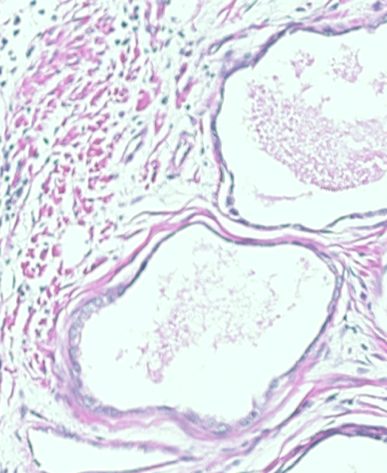

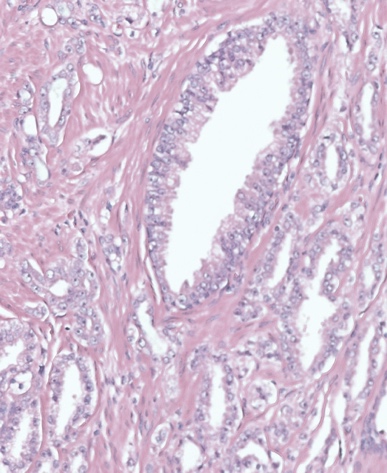

 (c) (d)

**Fig.S4. Scans of H&E stained regions after multispectral deep UV imaging. The rescanned regions are corresponding (a) Fig.2c (b) Fig.5c (c) Fig.5g (d) Fig.3c in the main text. It is clear that the tissue slices are not damaged and can be stained for further analysis.**

**References**

1. S. Soltani, A. Ojaghi, F. E. Robles, Deep UV dispersion and absorption spectroscopy of biomolecules. *Biomed. Opt. Express* **10**, 487-499 (2019).

2. P. Wormell, A. Rodger, in *Encyclopedia of Biophysics,* G. C. K. Roberts, Ed. (Springer Berlin Heidelberg, Berlin, Heidelberg, 2013), pp. 23-25.

3. S. Prasad, I. Mandal, S. Singh, A. Paul, B. Mandal, R. Venkatramani, R. Swaminathan, Near UV-Visible electronic absorption originating from charged amino acids in a monomeric protein. *Chemical Science* **8**, 5416-5433 (2017).

4. M. Schnell, S. Mittal, K. Falahkheirkhah, A. Mittal, K. Yeh, S. Kenkel, A. Kajdacsy-Balla, P. S. Carney, R. Bhargava, All-digital histopathology by infrared-optical hybrid microscopy. *Proceedings of the National Academy of Sciences* **117**, 3388 (2020).

5. S. Tiwari, K. Falahkheirkhah, G. Cheng, R. Bhargava, Colon Cancer Grading Using Infrared Spectroscopic Imaging-Based Deep Learning. *Appl. Spectrosc.* **76**, 475-484 (2022).

6. S. Mittal, T. P. Wrobel, M. Walsh, A. Kajdacsy-Balla, R. Bhargava, Breast cancer histopathology using infrared spectroscopic imaging: The impact of instrumental configurations. *Clinical Spectroscopy* **3**, 100006 (2021).

7. A. Mittal, S. Mittal, K. Yeh, A. Higham, R. Bhargava, in *Molecular and Laser Spectroscopy,* V. P. Gupta, Y. Ozaki, Eds. (Elsevier, 2020), pp. 595-622.

8. T. Matsumoto, H. Niioka, Y. Kumamoto, J. Sato, O. Inamori, R. Nakao, Y. Harada, E. Konishi, E. Otsuji, H. Tanaka, J. Miyake, T. Takamatsu, Deep-UV excitation fluorescence microscopy for detection of lymph node metastasis using deep neural network. *Scientific Reports* **9**, 16912 (2019).

9. C. Wong, M. E. Pawlowski, T. S. Tkaczyk, Simple ultraviolet microscope using off-the-shelf components for point-of-care diagnostics. *PLOS ONE* **14**, e0214090 (2019).

10. F. Fereidouni, Z. T. Harmany, M. Tian, A. Todd, J. A. Kintner, J. D. McPherson, A. D. Borowsky, J. Bishop, M. Lechpammer, S. G. Demos, R. Levenson, Microscopy with ultraviolet surface excitation for rapid slide-free histology. *Nature Biomedical Engineering* **1**, 957-966 (2017).

11. K. de Haan, Y. Zhang, J. E. Zuckerman, T. Liu, A. E. Sisk, M. F. P. Diaz, K.-Y. Jen, A. Nobori, S. Liou, S. Zhang, R. Riahi, Y. Rivenson, W. D. Wallace, A. Ozcan, Deep learning-based transformation of H&E stained tissues into special stains. *Nature Communications* **12**, 4884 (2021).

12. Y. Rivenson, H. Wang, Z. Wei, K. de Haan, Y. Zhang, Y. Wu, H. Günaydın, J. E. Zuckerman, T. Chong, A. E. Sisk, L. M. Westbrook, W. D. Wallace, A. Ozcan, Virtual histological staining of unlabelled tissue-autofluorescence images via deep learning. *Nature Biomedical Engineering* **3**, 466-477 (2019).

13. Y. J. Sepah, A. Akhtar, M. A. Sadiq, Y. Hafeez, H. Nasir, B. Perez, N. Mawji, D. J. Dean, D. Ferraz, Q. D. Nguyen, Fundus autofluorescence imaging: Fundamentals and clinical relevance. *Saudi J Ophthalmol* **28**, 111-116 (2014).

14. Y. Shen, F. Hu, W. Min, Raman Imaging of Small Biomolecules. *Annual Review of Biophysics* **48**, 347-369 (2019).

15. M. Uematsu, T. Shimizu, Raman microscopy-based quantification of the physical properties of intracellular lipids. *Communications Biology* **4**, 1176 (2021).

16. K. Czamara, F. Petko, M. Baranska, A. Kaczor, Raman microscopy at the subcellular level: a study on early apoptosis in endothelial cells induced by Fas ligand and cycloheximide. *Analyst* **141**, 1390-1397 (2016).
